# Associations between neighborhood built environment characteristics and cognitive function vary by area deprivation in a rural, ethnoracially diverse setting

**DOI:** 10.1002/alz70860_101246

**Published:** 2025-12-23

**Authors:** Lilah M Besser, Madeleine Tourelle, Diana Mitsova, Janet Holt, Lisa Ann Kirk Wiese, Christine L. Williams

**Affiliations:** ^1^ University of Miami Miller School of Medicine, Boca Raton, FL, USA; ^2^ Florida Atlantic University, Boca Raton, FL, USA; ^3^ C.E. Lynn College of Nursing, Florida Atlantic University, Boca Raton, FL, USA; ^4^ Florida Atlantic University, C.E. Lynn College of Nursing, Boca Raton, FL, USA

## Abstract

**Background:**

Studies have demonstrated that detrimental neighborhood environments (e.g., low greenspace access) are associated with poorer brain health outcomes including Alzheimer's disease and related dementia (ADRD) risk. However, these studies generally have focused on urban populations. Few studies have focused on rural populations and thus, evidence is limited on whether neighborhood built environments in rural, diverse communities benefit cognitive health.

**Methods:**

Using data from a NIA‐funded cohort in the rural Lake Okeechobee region of Florida, we investigated whether associations between built environment characteristics (by Census block group) and Montreal Cognitive Assessment (MoCA) scores vary by area deprivation level. We calculated neighborhood % open/park space using the National Land Cover Dataset, creating quartiles as prior studies suggest non‐linear greenspace‐brain health associations. Florida Geographic Data Library parcel data were used to calculate % retail space. Area deprivation index (ADI) values for each block group were dichotomized for analysis (ADI>90% (most deprived) versus ADI≤90%). Multivariable linear regression with generalized estimating equations (accounted for block group clustering) tested associations between % park/open space and retail space and MoCA scores, stratified by area deprivation. Models controlled for key demographics (e.g., age, gender, ethnoracial group, education (years)).

**Results:**

Participants (*n* = 384) were 64±10 years old; had 12±3 years of education; 72% were women; 77% were Black and 19% were White; and 16% were Hispanic. Mean MoCA scores were 25.3±4.1. Among those living in higher deprivation neighborhoods, individuals in the highest quartile of % open/park space had lower MoCA scores (Q4 vs Q1=‐2.70, 95% CI=‐3.75, ‐1.65) and those in neighborhoods with a greater % retail had higher MoCA scores (estimate=0.15, 95% CI=0.04, 0.25). Among those in lower deprivation neighborhoods, individuals in neighborhoods with the highest quartile of % open/park space had higher MoCA scores (Q4 vs Q1=3.50, 95% CI=2.37, 4.64) and those in neighborhoods with more retail had lower MoCA scores (estimate=‐0.35, 95% CI=‐0.45, ‐0.25).

**Conclusion:**

Among ethnoracially diverse older adults in rural Florida communities, associations between neighborhood % open/park space and retail space and cognitive function varied significantly by area deprivation.

